# Identification, expression, and association analysis of calcineurin B-like protein–interacting protein kinase genes in peanut

**DOI:** 10.3389/fgene.2022.939255

**Published:** 2022-09-05

**Authors:** Weifang Ren, Juncheng Zhang, Jie He, Jiahai Fang, Liyun Wan

**Affiliations:** ^1^ Key Laboratory of Crop Physiology, Ecology and Genetic Breeding, Ministry of Education, Jiangxi Agricultural University, Nanchang, China; ^2^ College of Life and Environmental Sciences, Hangzhou Normal University, Hangzhou, China

**Keywords:** peanut, expression, adversity response, association analysis, *CIPK* genes

## Abstract

Plants usually respond to the external environment by initiating a series of signal transduction processes mediated by protein kinases, especially calcineurin B-like protein–interacting protein kinases (CIPKs). In this study, 54 *CIPKs* were identified in the peanut genome, of which 26 were from cultivated species (named *AhCIPKs*) and 28 from two diploid progenitors (*Arachis duranensis—AdCIPKs* and *Arachis ipaensis—AiCIPKs*). Evolution analysis revealed that the 54 *CIPKs* were composed of two different evolutionary branches. The *CIPK* members were unevenly distributed at different chromosomes. Synteny analysis strongly indicated that whole-genome duplication (allopolyploidization) contributed to the expansion of *CIPK*. Comparative genomics analysis showed that there was only one common collinear *CIPK* pairs among peanut, *Arabidopsis*, rice, grape, and soybean. The prediction results of *cis*-acting elements showed that *AhCIPKs*, *AdCIPKs*, and *AiCIPKs* contained different proportions of transcription factor binding motifs involved in regulating plant growth, abiotic stress, plant hormones, and light response elements. Spatial expression profiles revealed that almost all *AhCIPKs* had tissue-specific expression patterns. Furthermore, association analysis identified one polymorphic site in *AdCIPK12* (*AhCIPK11*), which was significantly associated with pod length, seed length, hundred seed weight, and shoot root ratio. Our results provide valuable information of *CIPKs* in peanut and facilitate better understanding of their biological functions.

## Introduction

Calcium is an important second messenger in plants (Feng et al., 2017), and the signal transduction pathway mediated by calcium plays an important role in plant growth, development, and stress (Mao et al., 2016). Calcium signals formed during plant growth are further transmitted by calcium sensor proteins. Common calcium sensor proteins include calmodulins (CaMs), calmodulin-like proteins (CMLs), calcium-dependent protein kinases (CDPK), calcineurin B-like proteins (CBL), and CBL-interacting protein kinase (CIPK) (Zhao et al., 2009). CIPK, the interacting protein kinase of CBL, is a kind of protein that interacts with activated CBL as a downstream protein and is also a kind of Ca^2+^-dependent serine/threonine protein kinase (Kim et al., 2000), which contains a conserved catalytic kinase domain at its N-terminal (Albrecht et al., 2001). The C-terminal is an NAF regulatory control domain composed of 24 amino acids, which is highly conserved and mediates the interaction between CBL and CIPK protein (Kolukisaoglu et al., 2004).

Up to now, 25, 30, 16, and 43 *CIPK* genes have been identified in *Arabidopsis thaliana* (Yu et al., 2007), *Oryza sativa* (Kolukisaoglu et al., 2004), *Vitis vinifera* (Lu et al., 2017), and *Zea mays* (Chen et al., 2011), respectively. *CIPK* genes were widely involved in stress response, growth, and development regulation of plants (Hu et al., 2015). Studies of *Arabidopsis* have shown that the protein encoded by *AtCIPK23* played an important role in potassium metabolism (Wang and Wu, 2009). The combination of *AtCBL1*, *AtCBL9*, and *AtCIPK23* activated the potassium transport channel (Cheong et al., 2007). AtCIPK15 interacted with PP2C phosphatase ABI2 to form a complex, which controlled the expression of ABA-related genes (Li et al., 2006). *AtCIPK3* responded to various abiotic stresses through ABA-dependent or ABA-independent pathway (Xu et al., 2006). It was found that overexpression of *OsCIPK3*, *OsCIPK12*, and *OsCIPK15* improved cold, drought, and salt stress tolerance of rice, respectively (Xiang et al., 2007). Overexpression of wheat *CIPK24* increased the contents of Na^+^ and antioxidant protective enzymes (Hrabak et al., 2003) and further improved the tolerance of *A. thaliana* to high salt stress (Deng et al., 2013). Apple *CIPK6* interacted with *AtCBL4* protein after transferring *A. thaliana AtCBL4* gene into apple and improved the resistance of apple seedlings to low temperature, drought, and high salt stress (Wang and Liu, 2018). With the completion of large-scale plant genome sequencing, the research on *CIPK* genes in soybean (Feng et al., 2015), poplar (Sun et al., 2015), and many other plants received considerable attention, but research on *CIPK* genes in peanut has not been reported yet.

Peanut is one of the major oil and cash crops in China (Pandey et al., 2020). Calcium is the second largest nutrient element in peanut (Rui, 2015). In recent years, frequent occurrence of land drought, cold damage, and soil salinization has been a serious impact on the increase of peanut yield and the improvement of peanut quality (Lu et al., 2017). Under the pressure of reducing production cost and protecting the environment, screening peanut varieties with low calcium tolerance and exploring multistress response proteins and stress resistance mechanisms in an adverse environment have become the top priority in studying the stress resistance breeding of peanut (Sanders et al., 2002). To deal with abiotic stresses such as drought, cold, and salt damage and improve the yield and quality of peanut, it is of great significance to explore peanut *CIPK* genes and reveal their role in the calcium signaling pathway. In this study, the *CIPK* genes of cultivated peanut and its two diploid progenitors were comprehensively analyzed. At last, a total of 54 *CIPK* genes were explicitly identified. Their basic protein information, exon–intron structure, phylogeny, and *cis*-acting elements were systematically analyzed, which provided valuable theoretical basis and genetic resources for the high-yield breeding of peanut.

## Materials and methods

### Genome-wide identification of the *CIPK* genes in peanut

To identify *CIPK* genes in peanut, the protein sequences of three peanut genomes were downloaded from PeanutBase (http://www.peanutbase.org/). The conserved domains of all proteins encoded by peanut genome were analyzed using the HMMER 3.0 software, and genes including both the Pkinase (PF00069.24) and NAF (PF03822.13) domains were selected as peanut *CIPK* candidates. The PROSTIE and SMART software were used to verify the 54 CIPKs as calcineurin B-like protein–interacting protein kinases. Candidates with PROTEIN_KINASE_DOM (PS50011) and NAF (PS50816) in PROSTIE and the S_TKc (SM00220) domain in SMART were selected as CIPKs. The physicochemical data such as gene number, coding sequence (CDS) length, amino acid number, isoelectric point, molecular weight, and EF hand structure number were obtained from PeanutBase or analyzed using the ExPASy Proteomics Sever online tool (Gasteiger et al., 2005).

### Evolution and structure analysis of *CIPK* genes in peanut

The gene structure diagram was drawn using the GSDS 2.0 mapping software based on the genome annotation information of *CIPK* genes with the GFF format, which was downloaded from PeanutBase (http://www.peanutbase.org/). The phylogenetic tree using 179 CIPK proteins from peanut, rice, grape, *Arabidopsis*, and soybean was constructed using the MEGA 5.2 software with the neighbor-joining method (Bootstrap value 1,000, Poisson model, uniform rates, pairwise deletion). The analyses of the composition of conserved motifs were conducted using MEME (http://meme-suite.org/tools/meme) with the maximum number 20 (classic mode, zero or one occurrence per sequence).

### *Cis*-acting elements analysis of peanut *CIPKs*

We defined the 2-kb upstream sequence of the initiation codon as the promoter of the peanut *CIPKs* and downloaded it from PeanutBase to search for *cis*-acting regulatory elements through PlantCARE (http://bioinformatics.psb.ugent.be/webtools/plantcare/html/). Then, only the *cis*-acting elements related to adversity stress were screened out statistically.

### Expression profiles of *AhCIPK* genes in different tissues and treatments

RNA-seq datasets of 22 peanut tissues were downloaded from PeanutBase (http://www.peanutbase.org/) and NCBI SRA (https://www.ncbi.nlm.nih.gov/sra/), and the expression levels of *AhCIPK* genes in different tissues were obtained (Clevenger et al., 2016) with all raw data deposited as BioSamples SAMN03944933–SAMN03944990. The expression data (FPKM value) of peanut *CIPK* genes were normalized and output using the TBtools software (Chen et al., 2020). *Ralstonia solanacearum* infection was carried out as described before according to (Zhang et al. 2017). Submergence treatment followed the method described by (Zeng et al. 2021).

### Candidate gene association mapping

The genotype data of the *CIPK* genes used for association analyses were obtained from the transcriptome sequencing data of a peanut germplasm population with 146 accessions (unpublished data). The phenotypes were collected from five environments (Wuhan 2016, Wuhan 2017, Yangluo 2016, Yangluo 2017, Zhanjiang 2016). Three replicates were randomly planted in each environment, with 12 plants in each row.

## Results

### Genome-wide identification of the *CIPK* genes in cultivated peanut and its diploid progenitors

**FIGURE 1:**
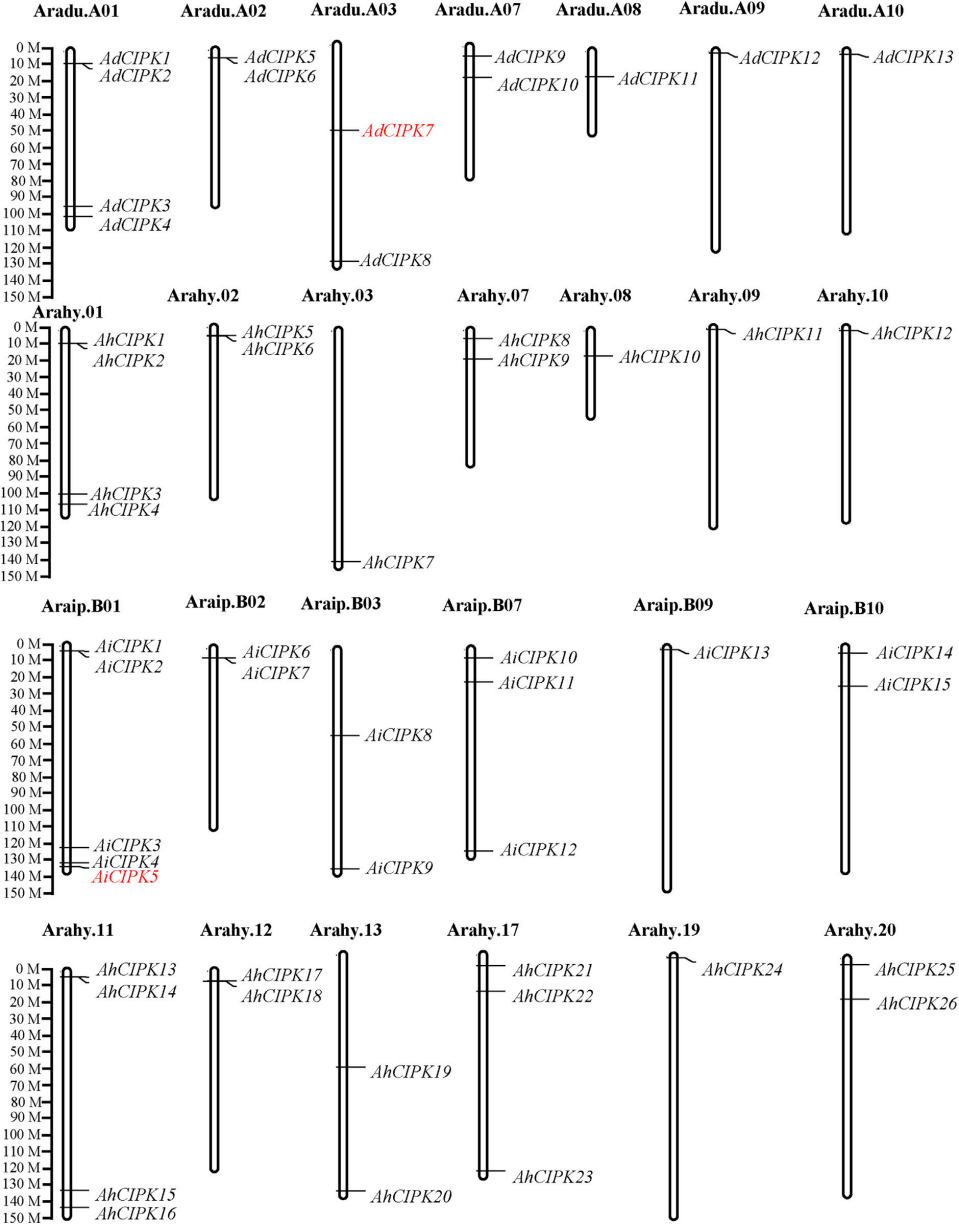
Chromosomal locations of peanut calcineurin B-like protein–interacting protein kinase (*CIPK*) genes. Chromosomal positions of the peanut *CIPK* genes were mapped based on GFF data downloaded from PeanutBase. The chromosome number is indicated above each chromosome. Genes in red mean wild species specific.

To systematically determine *CIPK* genes in peanut, genes containing both the conserved Pkinase (PF00069.24) and NAF (PF03822.13) domains were searched through the whole peanut genome. The SMART and PROSTIE software tools were used to verify the Pkinase domains. A total of 54 *CIPK* candidates were identified from the peanut genome of cultivated species *Arachis hypogaea* (26, namely *AhCIPK1*–*AhCIPK26*) and its two wild species *Arachis duranensis* (13, namely, *AdCIPK1*–*AdCIPK13*) and *Arachis ipaensis* (15, namely, *AiCIPK1*–*AiCIPK15*) (Table 1). Then, we determined their chromosome locations, mRNA length, number of amino acids (aa), MW, theoretical pI, and transmembrane domain (TMD) (Table 1). *AhCIPK* genes were distributed on chromosomes 1, 2, 3, 7, 8, 9, and 10 (A genome) and 11, 12, 13, 17, 19, and 20 (B genome). Two wild species–specific *CIPK* genes (*AdCIPK7* and *AiCIPK5*) were uncovered (Figure 1). The gene length of the peanut *CIPKs* ranged from 1,146 to 3,177 bps, of which the shortest was *AiCIPK4* with 1,146 bps and the longest length was *AhCIPK22* with 3,177 bps. The amino acid length of *CIPKs* varied from 381 to 570. The isoelectric point ranged from 5.8 (*AdCIPK11*) to 9.79 (*AiCIPK8*), and the molecular weight ranged from 45,067.06 to 64,189.82 Da. All CIPKs do not contain a TMD.

**TABLE 1:** Information on CIPK genes identified in peanuts.

Gene name	Gene locus	CDS length (bp)	AA^a^	MW^b^ (kDa)	pI^c^	TMD^d^	Chr
*AdCIPK1*	*Aradu.9W61Z*	1,386	461	51.86	8.38	0	Aradu.A01
*AdCIPK2*	*Aradu.TL55R*	1,582	453	51.10	8.48	0	Aradu.A01
*AdCIPK3*	*Aradu.K8K3S*	2002	463	52.10	8.64	0	Aradu.A01
*AdCIPK4*	*Aradu.SX4F9*	1,670	452	50.61	8.35	0	Aradu.A01
*AdCIPK5*	*Aradu.MRA83*	1,645	434	49.26	8.44	0	Aradu.A02
*AdCIPK6*	*Aradu.T9ESX*	1,221	406	46.38	8.80	0	Aradu.A02
*AdCIPK7*	*Aradu.HS592*	2,199	457	51.51	9.22	0	Aradu.A03
*AdCIPK8*	*Aradu.8B2M9*	2,311	466	51.93	8.84	0	Aradu.A03
*AdCIPK9*	*Aradu.73JAV*	1982	441	50.46	6.42	0	Aradu.A07
*AdCIPK10*	*Aradu.7W2Z9*	2,110	456	50.40	8.30	0	Aradu.A07
*AdCIPK11*	*Aradu.V638G*	1,378	457	51.72	5.85	0	Aradu.A08
*AdCIPK12*	*Aradu.Z7XZ9*	1,681	461	52.41	8.75	0	Aradu.A09
*AdCIPK13*	*Aradu.Q5XDE*	1,653	550	61.66	8.17	0	Aradu.A10
*AiCIPK1*	*Araip.L2Z00*	1,582	455	51.24	8.06	0	Araip.B01
*AiCIPK2*	*Araip.X0WZQ*	1,386	461	51.93	8.38	0	Araip.B01
*AiCIPK3*	*Araip.I6C5W*	1,591	427	47.72	8.37	0	Araip.B01
*AiCIPK4*	*Araip.J6DER*	2,212	456	51.33	8.09	0	Araip.B01
*AiCIPK5*	*Araip.MS6UX*	1,146	381	43.51	8.81	0	Araip.B01
*AiCIPK6*	*Araip.A3V01*	1,239	412	46.65	8.52	0	Araip.B02
*AiCIPK7*	*Araip.CMC8E*	1,317	413	47.25	6.54	0	Araip.B02
*AiCIPK8*	*Araip.B16CX*	1,663	508	57.37	9.79	0	Araip.B03
*AiCIPK9*	*Araip.M3K7N*	2,359	465	51.84	8.84	0	Araip.B03
*AiCIPK10*	*Araip.7IS5A*	1991	441	50.46	6.42	0	Araip.B07
*AiCIPK11*	*Araip.WP1GX*	2021	456	50.40	8.02	0	Araip.B07
*AiCIPK12*	*Araip.Z7THM*	2,259	452	51.10	6.72	0	Araip.B07
*AiCIPK13*	*Araip.KS6V8*	1,677	461	52.56	8.49	0	Araip.B09
*AiCIPK14*	*Araip.W1LHP*	1,653	550	61.63	8.17	0	Araip.B10
*AiCIPK15*	*Araip.882H9*	1,387	436	48.93	9.00	0	Araip.B10
*AhCIPK1*	*Arahy.TE3LXI*	1,386	461	51.86	8.38	0	Arahy.01
*AhCIPK2*	*Arahy.N6YX8I*	2,735	453	51.10	8.48	0	Arahy.01
*AhCIPK3*	*Arahy.069RBA*	2014	446	50.44	8.79	0	Arahy.01
*AhCIPK4*	*Arahy.HIWA31*	3,017	570	64.19	9.22	0	Arahy.01
*AhCIPK5*	*Arahy.MA0DIS*	2,252	448	50.59	9.21	0	Arahy.02
*AhCIPK6*	*Arahy.T0XBGT*	1,516	398	45.07	9.31	0	Arahy.02
*AhCIPK7*	*Arahy.9ML3HZ*	2,762	497	55.49	8.90	0	Arahy.03
*AhCIPK8*	*Arahy.KQQ5DM*	1,218	405	46.26	6.26	0	Arahy.07
*AhCIPK9*	*Arahy.50QZS1*	2,813	456	50.40	8.30	0	Arahy.07
*AhCIPK10*	*Arahy.YEIN47*	2,295	549	61.97	7.56	0	Arahy.08
*AhCIPK11*	*Arahy.R30FAJ*	2,874	461	52.41	8.75	0	Arahy.09
*AhCIPK12*	*Arahy.L0AIUK*	1,653	550	61.66	8.17	0	Arahy.10
*AhCIPK13*	*Arahy.RMG3R2*	1,365	454	51.14	8.18	0	Arahy.11
*AhCIPK14*	*Arahy.M8C26Q*	1,386	461	51.90	8.38	0	Arahy.11
*AhCIPK15*	*Arahy.4 × 641H*	2,976	501	56.15	8.66	0	Arahy.11
*AhCIPK16*	*Arahy.QIVE9X*	2015	446	50.44	8.79	0	Arahy.11
*AhCIPK17*	*Arahy.I9I2G1*	1948	417	47.05	8.97	0	Arahy.12
*AhCIPK18*	*Arahy.LMT476*	1,318	427	48.48	9.13	0	Arahy.12
*AhCIPK19*	*Arahy.84D15R*	1,365	454	51.18	8.79	0	Arahy.13
*AhCIPK20*	*Arahy.JQ1SFF*	2,694	465	51.84	8.84	0	Arahy.13
*AhCIPK21*	*Arahy.EY5MJ2*	1,218	405	46.26	6.26	0	Arahy.17
*AhCIPK22*	*Arahy.42B8G7*	2,818	456	50.40	8.02	0	Arahy.17
*AhCIPK23*	*Arahy.9NB62H*	3,177	492	55.53	6.95	0	Arahy.17
*AhCIPK24*	*Arahy.XP1WSF*	2,874	461	52.56	8.49	0	Arahy.19
*AhCIPK25*	*Arahy.VY9V4D*	1,653	550	61.63	8.17	0	Arahy.20
*AhCIPK26*	*Arahy.D01KFK*	1,361	436	48.93	9.00	0	Arahy.20

a Length of the amino acid sequence.

b Molecular weight of the amino acid sequence.

c Isoelectric point of the AhCIPKs.

d Number of transmembrane domains, as predicted by the TMHMM Server v2.0.

CDS, coding sequence.

### Phylogenetic and gene structure analysis of *CIPKs* in peanut

**FIGURE 2:**
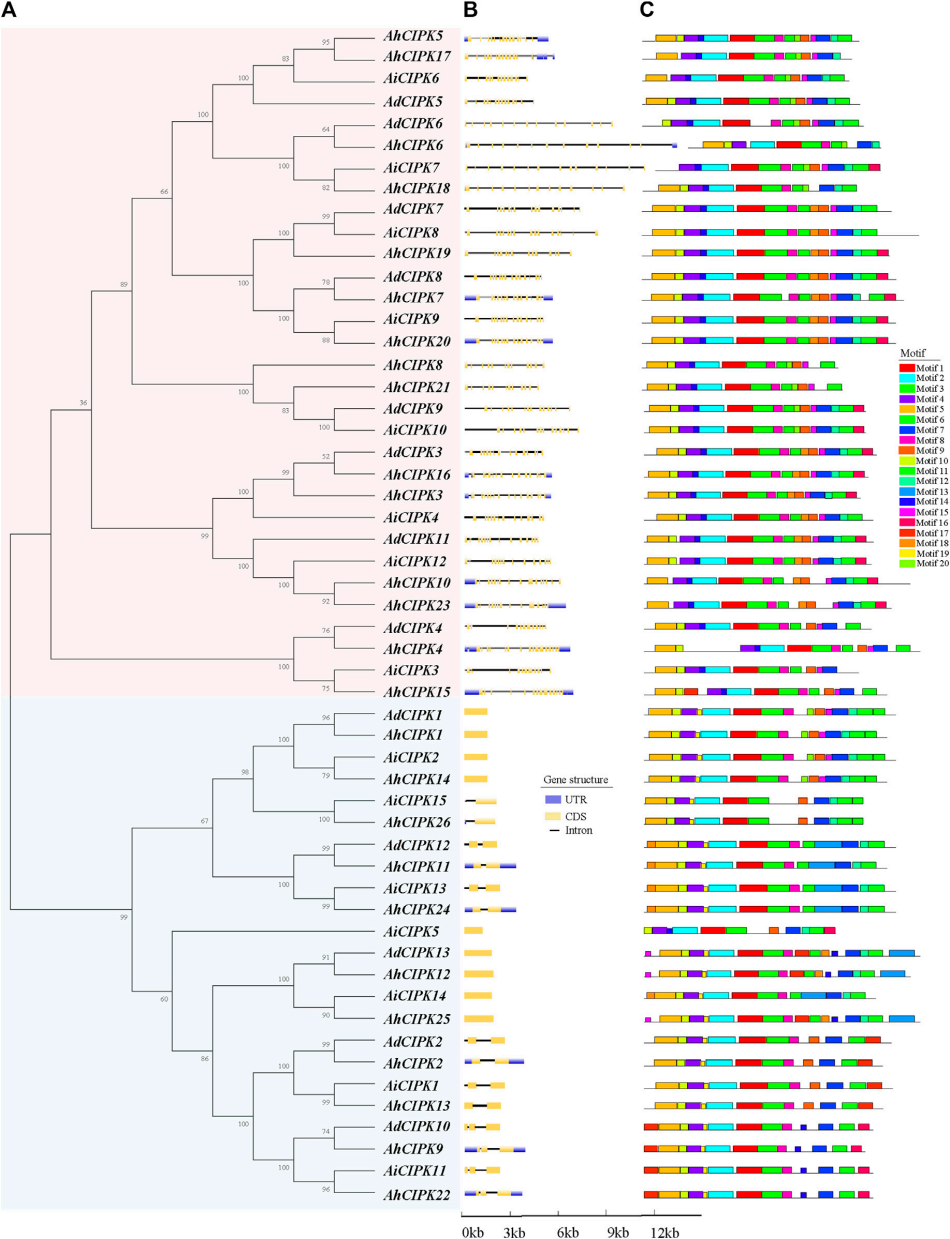
Comparison of the gene structure and motif of 54 *CIPK* genes in peanut. **(A)** unrooted phylogenetic tree with over 50% bootstrap value above the branch. Clades I and II are displayed in pink and blue colors, respectively. The names of the species are abbreviated to two letters, named as *Arachis duranensis* (Ad), *Arachis ipaensis* (Ai), and *Arachis hypogaea* L. *Tifrunner* (Ah). **(B)** exon/introns and untranslated regions (UTRs) of *CIPK*s. Blue boxes denote UTR (untranslated region); yellow boxes denote coding sequence (CDS); and black lines denote introns. The length of protein can be estimated using the scale at the bottom. **(C)** motif architectures of all *CIPK* genes. Each motif is illustrated with a specific color, and the distribution of identified motifs corresponds to their positions.

To determine the evolutionary relationship of *CIPKs* among *A. hypogaea*, *A. duranensis*, and *A. ipaensis*, the phylogenetic tree of the 54 *CIPKs* was constructed. The results indicated that the *CIPKs* can be classified into two clades (I and Ⅱ) (Figure 2A). Clades I and II consisted of 31 *CIPKs* (15 *AhCIPKs*, 8 *AdCIPKs*, and 8 *AiCIPKs*) and 23 *CIPKs* (11 *AhCIPKs*, 5 *AdCIPKs*, and 7 *AiCIPKs*), respectively. It is interesting that the results of the gene structure based on the genome annotations also showed that the *CIPK* genes can be divided into two groups, corresponding to the two phylogenetic families (the intron-rich group corresponded to phylogenetic clade I, and the intron-less group corresponded to phylogenetic clade Ⅱ). The intron numbers of the intron-less group were less than 3 (0 to 2), while those of the intron-rich group were more than 10 (Figure 2B). Further, the phylogenetic relationship and classification of peanut CIPKs were supported by motif analysis. A total of 20 motifs were identified (Figure 2C); in general, peanut CIPKs had 11–17 motifs. Motif 1, Motif 2, and Motif 4 were the most common, present in all CIPK proteins. Otherwise, the vast majority of *CIPKs* included Motifs 3, 5, 6, 7, and 8, which covered more than 50 CIPK members. Motifs 13 and 19 were clade-specific elements in clade II, and Motif 17 only existed in AhCIPK15 of clade I but 13 CIPKs in clade II.

### Biological evolution analysis of CIPKs in peanut and other plant species

**FIGURE 3:**
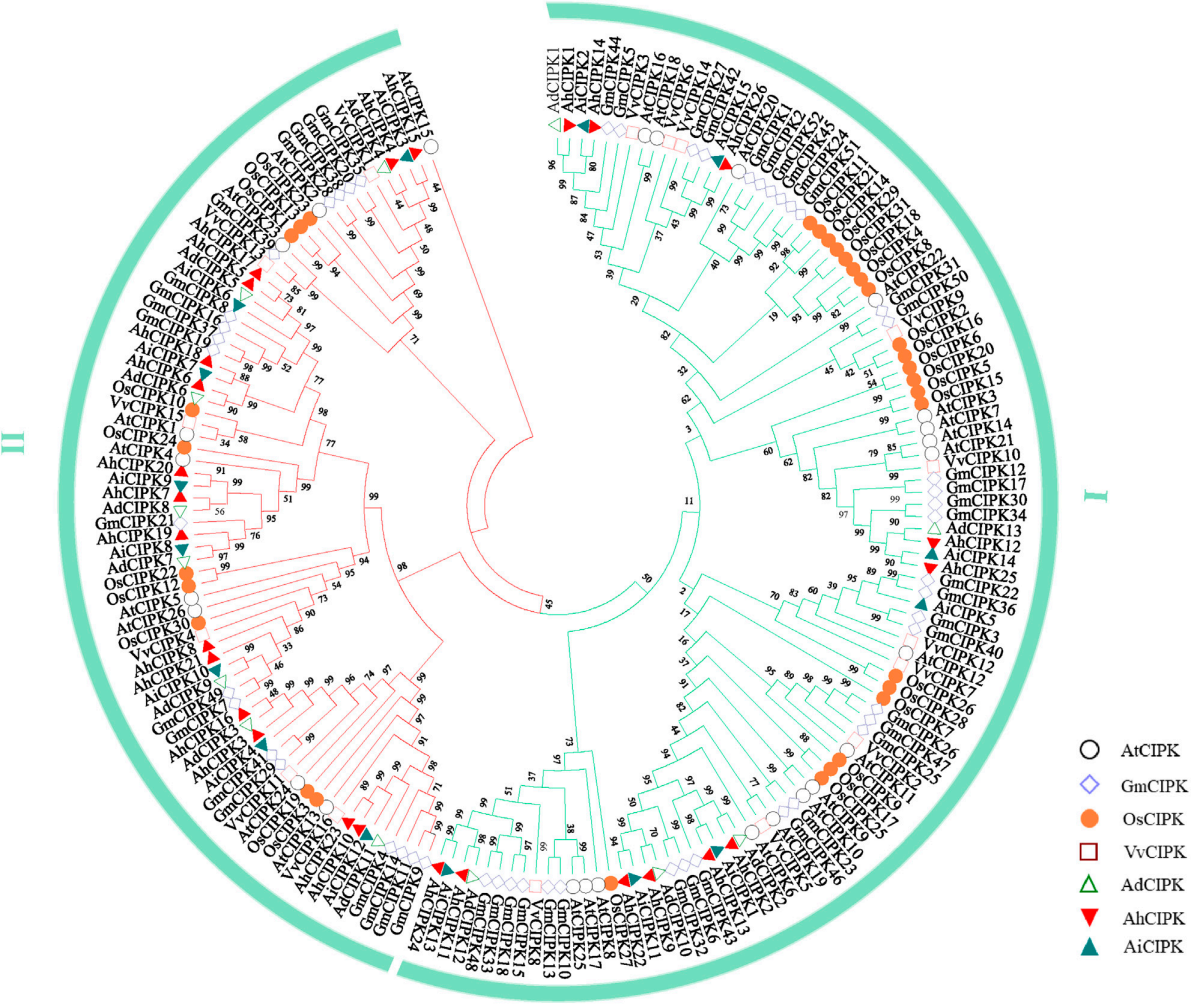
Phylogenetic tree of CIPKs in peanut and other plant species. The plants in the square frame indicate that the CIPK members outside of peanuts have the closest evolutionary relationship with soybean CIPKs.

To further understand the relationship of CIPK members among different species, the phylogenetic tree of CIPK proteins of *Arabidopsis*, rice, grape, soybean [AtCIPK (26), OsCIPK (31), VvCIPK(16), and GmCIPK (52)], and peanut was constructed using maximum parsimony (Figure 3). The results showed that *CIPK* proteins of these species can be divided into two subfamilies (Ⅰ and Ⅱ). The analysis of the phylogenetic tree revealed that all the peanut CIPKs were clustered together (Figure 3). The relationships between the two wild species *A. duranensis* and *A. ipaensis* and cultivated species *A. hypogaea* were closer than that between the other four species. In addition, many CIPK members of peanut and soybean clustered together, suggesting that the two legume species were evolutionarily closer than others. TThe second evolutionary closest of peanut was *Arabidopsis*. There were more family members in peanut and soybean than in *Arabidopsis*, rice, and grape plants, suggesting a specific linear amplification of the gene family in legume plant. Whether these additional members of the genes have additional functions as well or whether they are produced only because functional redundancy requires further experimental verification.

### Gene duplication and synteny analyses of peanut *CIPKs*

**FIGURE 4:**
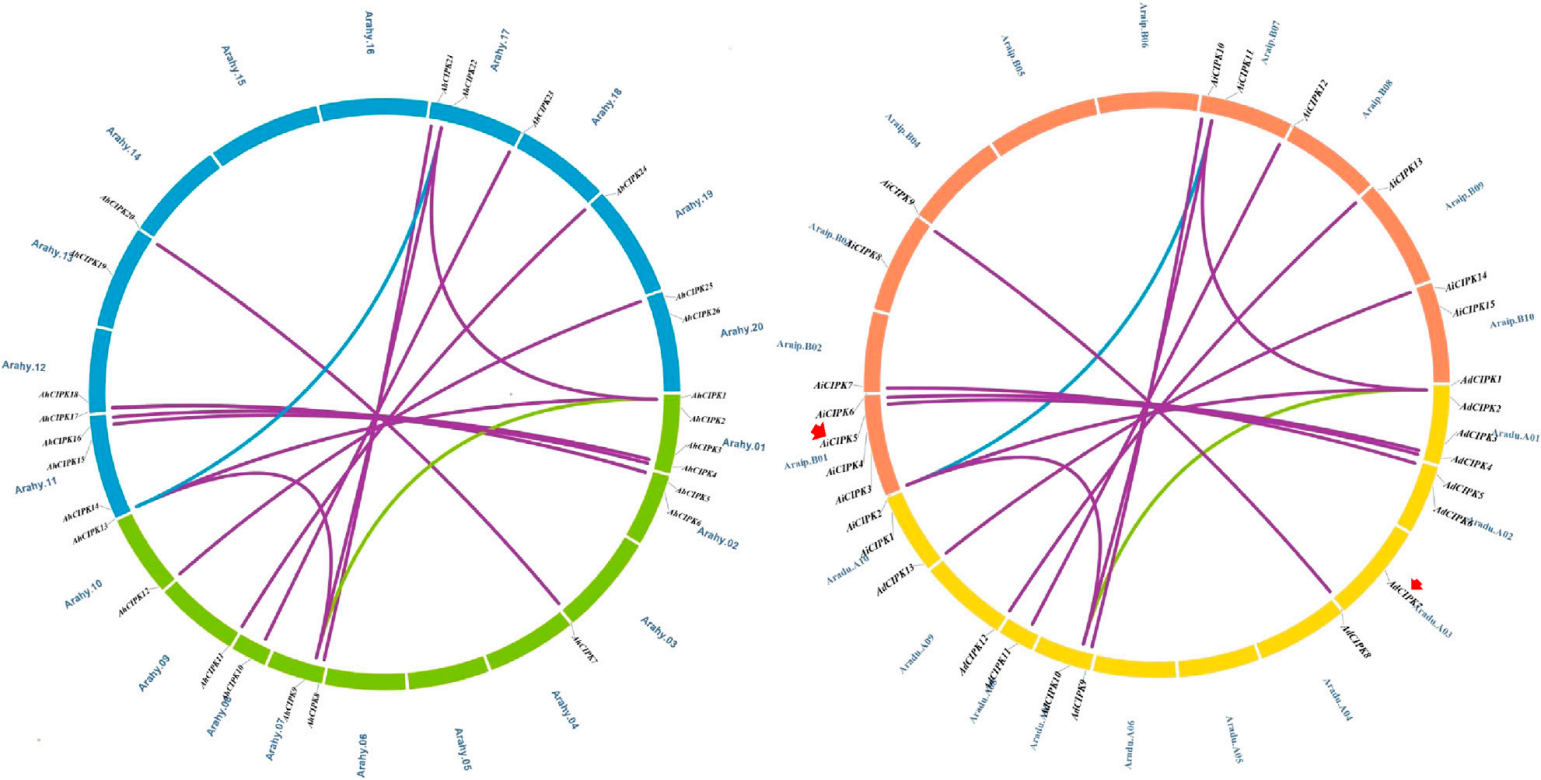
Chromosomal distribution and gene duplications of the *AdCIPK*, *AiCIPK*, and *AhCIPK* genes. The scales on the circle are in megabases. Each colored bar represents a chromosome as indicated. Gene IDs are labeled on the basis of their positions on the chromosomes. Red arrows indicate wild species–specific *CIPK* genes.

**FIGURE 5:**
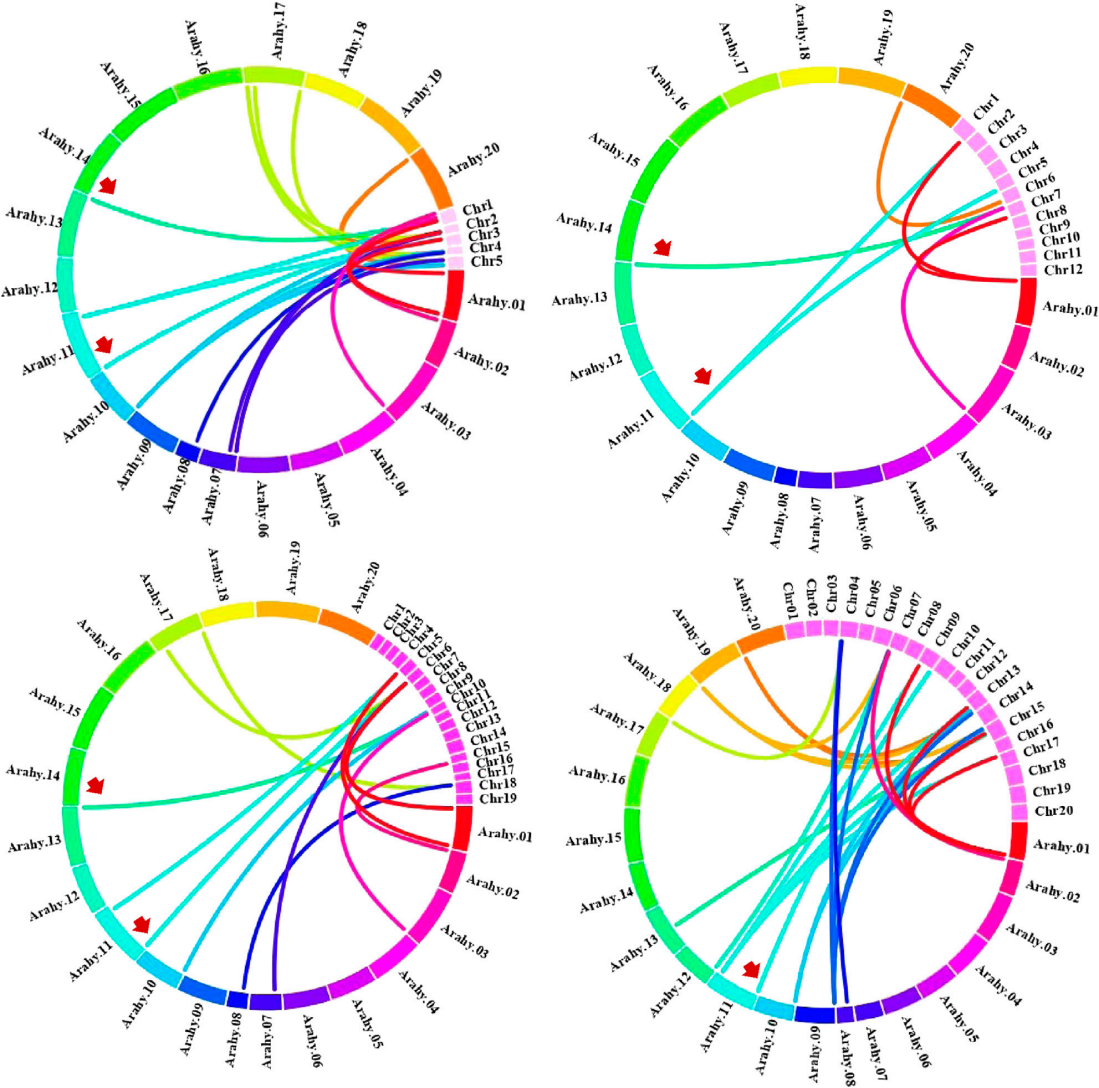
Comparative physical mapping showed orthologous relationships of AhCIPK genes with (**A**) *Arabidopsis,* (**B**) rice, (**C**) grape, and (**D**) soybean. Red arrows indicate common collinear *CIPK* orthologs.

Chromosomal location analyses revealed that the 26 *AhCIPKs* distributed unevenly on 13 chromosomes (chromosomes 01, 02, 03, 07, 08, 09, 10, 11, 12, 13, 17, 19, and 20). The 13 *AdCIPK*s presented on chromosomes A01, A02, A03, A07, A08, A09, and A10, and the 15 *AiCIPK*s distributed on chromosomes B01, B04, B06, B09, and B10 (Figure 4). A total of 16 chromosomal fragment repeat gene pairs were identified without tandem repeats (Figure 4, Supplementary Table S1). Further, we calculated the Ks (synonymous) and Ka (nonsynonymous) values of the duplicated gene pairs and found that the Ka/Ks ratio for duplicated *AhCIPK* gene pairs ranged from 0.00 to 0.57 with an average of 0.17 (Supplementary Table S2). The *ω* values of all duplicated gene pairs were less than 1, showing that purifying selection occurred on these duplicated gene pairs. Synteny analysis with *Arabidopsis*, rice, grape, and soybean revealed one conserved *CIPK* gene (*AhCIPK14*) in these species (Figure 5, Supplementary Table S1). BLASTP methods were used to identify peanut *CIPK* gene orthologs between peanut and *Arabidopsis*. In total, we found 54 orthologous gene pairs between peanut and *Arabidopsis* (Table 2). The orthologs in *Arabidopsis* included *AtCIPK12/AtWL4* and *AtCIPK5* participating in pollen germination and tube growth (Wang et al., 2008; Steinhorst et al., 2015), *AtCIPK24/AtSOS2* required for salt tolerance in *A. thaliana* (Halfter et al., 2000; Ishitani et al., 2000; Liu et al., 2000; Guo et al., 2001), and *AtCIPK1* and *AtCIPK3* relating to the ABA signal transduction (D'Angelo et al., 2006; Sanyal et al., 2017; Pandey et al., 2008; Kim et al., 2003). Therefore, we speculated that these *AhCIPK* homologous genes might play multiple roles not only in peanut growth and development but also in plant hormone and stress resistance.

**TABLE 2:** The function of AhCIPKs genes homologous to *Arabidopsis*.

Peanut	*Arabidopsis*	Function	References
*AdCIPK4/AiCIPK3/AhCIPK4/AhCIPK15*	*AtCIPK1* (*At3G17510*)	Controls abscisic acid-dependent and independent stress responses	D'Angelo et al. (2006)
*AdCIPK5/AdCIPK6/AiCIPK6/AiCIPK7/AhCIPK5/AhCIPK6/AhCIPK17/AhCIPK18*	*AtCIPK9* (*At1G01140*)	A calcium sensor-interacting protein kinase required for low-potassium tolerance	Pandey et al. (2007)
			Singh et al. (2018)
			Lara et al. (2020)
			Kanwar et al. (2022)
*AdCIPK7/AdCIPK8/AiCIPK8/AiCIPK9/AhCIPK7/AhCIPK19/AhCIPK20*	*AtCIPK23* (*At1G30270*)	Serves as a positive regulator of the potassium transporter AKT1 by directly phosphorylating AKT1	Sánchez-Barrena et al. (2020)
			Ragel et al. (2015)
			Tian et al. (2016)
			Wang et al. (2016)
			Xu et al. (2006)
			Cheong et al. (2007)
*AhCIPK8/AhCIPK21/AdCIPK9/AiCIPK10*	*AtCIPK3* (*At2G26980*)	Regulates Abscisic Acid and Cold Signal Transduction	Sanyal et al. (2017)
			Pandey et al. (2008)
			Kim et al. (2003)
*AdCIPK2/AdCIPK10/AiCIPK1/AiCIPK11/AhCIPK2/AhCIPK9/AhCIPK13/AhCIPK22/*	*AtCIPK11* (*At2G30360*)	A positive regulator in cadmium stress response	Zhou et al. (2015)
			Gu et al. (2021)
			Liu and Guo, (2011)
*AdCIPK13/AiCIPK14/AhCIPK12/AhCIPK25*	*AtCIPK12/AtWL4* (*At4G18700*)	Required for Polarized Pollen Tube Growth	Steinhorst et al. (2015)
*AdCIPK11/AiCIPK12/AhCIPK10/AhCIPK23*	*AtCIPK8* (*At4G24400*)	Regulates the low-affinity phase of the primary nitrate response	Hu et al. (2009)
			Gong et al. (2002)
*AiCIPK5*	*AtCIPK6* (*At4G30960*)	Required for development and salt tolerance	Sardar et al. (2017)
			Chen et al. (2013)
			Tsou et al. (2012)
			Held et al. (2011)
			Tripathi et al. (2009a)
			Tripathi et al. (2009b)
*AdCIPK12/AiCIPK13/AhCIPK11/AhCIPK24*	*AtCIPK5* (*At5G10930*)	Regulates potassium homeostasis under low oxygen	Schlücking et al. (2013)
			Tagliani et al. (2020)
*AdCIPK3/AiCIPK4/AhCIPK3/AhCIPK16*	*AtCIPK24/AtSOS2* (*At5G35410*)	SOS2 gene encodes a protein kinase that is required for salt tolerance	Liu et al. (2000)
			Halfter et al. (2000)
			Ishitani et al. (2000)
			Guo et al. (2001)
*AdCIPK1/AiCIPK2/AiCIPK15/AhCIPK1/AhCIPK14/AhCIPK26*	*AtCIPK5* (*At5G58380*)	Gene expression to accompany pollen germination and tube growth	Wang et al. (2008)

### *Cis*-acting elements prediction of *CIPK* genes in peanut

**FIGURE 6:**
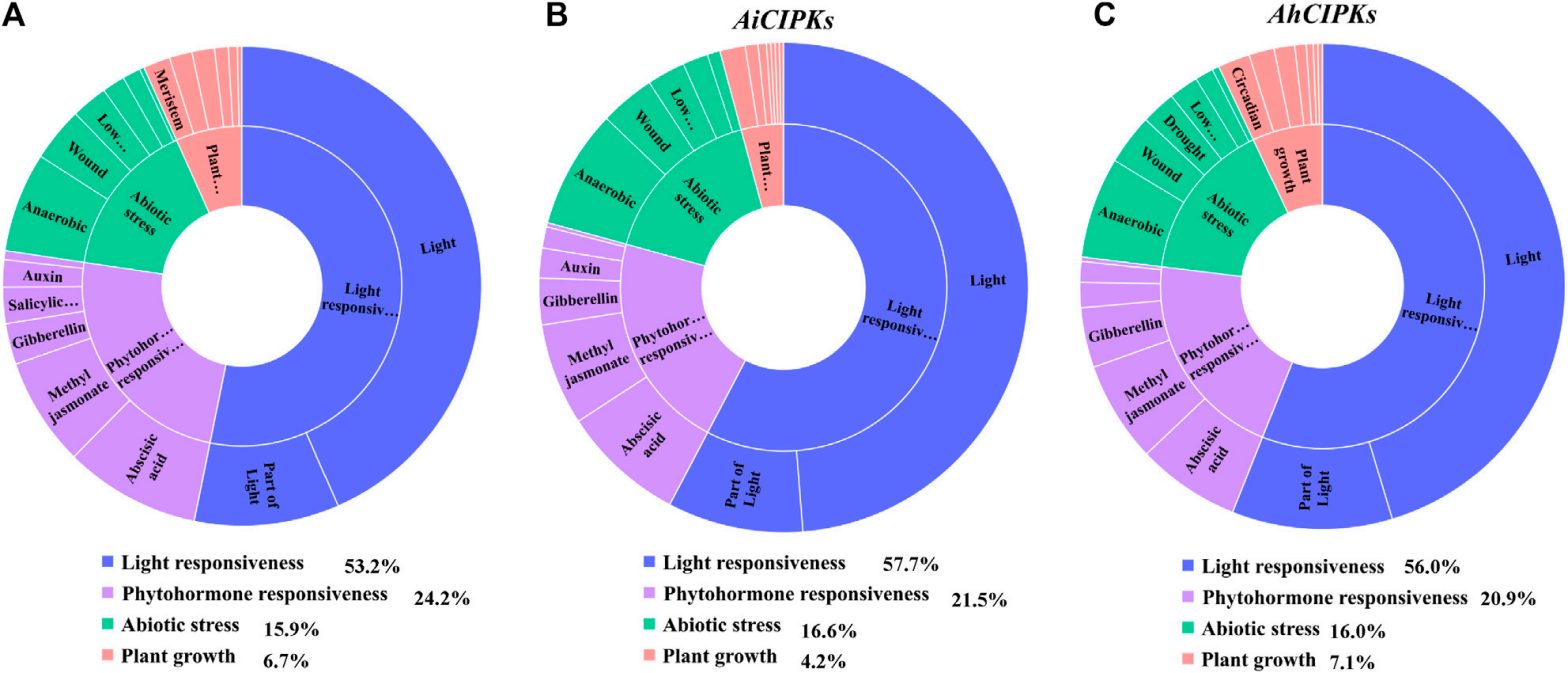
*cis*-acting elements in peanut *CIPK* genes. **(A)** assessment of *AdCIPK* different subclass and category proportions in sunburst chart. **(B)** assessment of *AiCIPK* different subclass and category proportions in sunburst chart. **(C)** assessment *AhCIPK* different subclass and category proportions in sunburst chart. The lengths of the petals are proportional to the number of elements in each subclass qualitatively. Blue, purple, green, and red represent light responsiveness, phytohormone responsiveness, abiotic stress responsiveness, and plant growth regulation, respectively.

*Cis*-acting elements in a promoter as the binding target of transcription factors are essential in the regulation of gene expression. In order to understand the regulation mechanisms of peanut *CIPK* genes, 2-kb upstream sequences of the peanut *CIPK* genes were analyzed via the PlantCARE database. In total, 54 *cis*-regulatory elements were detected. Four main categories were defined as the light responsiveness element, phytohormone responsiveness, abiotic stress responsiveness, and plant growth groups (Figures 6A–C, Supplementary Table S3). In the promoter region of the *AdCIPKs*, the largest subdivision was the light responsiveness group, containing 53.2% of the predicted *cis*-elements; phytohormone responsiveness elements ranked second (24.2%) (Figure 6A); abiotic stress response elements were 15.9%; and elements involved in plant growth accounted for 6.7% (Figure 6A). *AdCIPK2* had the greatest number of elements with 38 in total, which contained six abscisic acid responsiveness elements (ABREs) (Supplementary Table S3). For *AiCIPKs*, the percentage of light, phytohormone, abiotic stress, and plant growth responsiveness *cis*-elements was 57.7, 21.5, 16.6, and 4.2% (Figure 6B). *AiCIPK14* had the greatest number of elements at 40 in total, which also contained six ABREs. In *AhCIPKs*, the proportions were 56.0, 20.9, 16.0, and 7.1% (Figure 6C). In the light response category, Box 4 (light-responsive element) and GT1-motif (part of a module for light response) were the most dominant. Meanwhile, *cis*-acting elements responding to auxin, abscisic acid, gibberellin, flavonoids, methyl jasmonate, and salicylic acid were detected in the phytohormone responsiveness group.

### Tissue expression profiles of *AhCIPKs*

**FIGURE 7:**
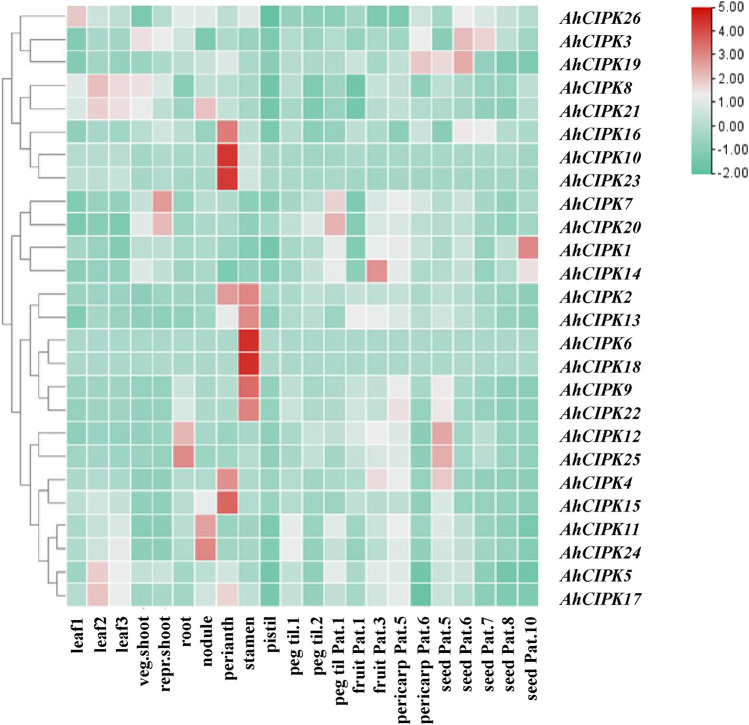
Expression profiles of *AhCIPK* genes. The heatmap of the *AhCIPK* gene expression levels was hierarchically clustered using TBtools with the data normalization method of Z-score standardization. The color scale bar from green to red represents low and high expressions, respectively. Abbreviations of the 22 tissues used in the expression profiles of *AhCIPK* genes were from Ren et al. (2022).

To further study the expression pattern of peanut *CIPKs* in different tissues and explore its function in peanut growth and development, the tissue expression profiles of *CIPK* genes were analyzed by using the transcriptome data of 22 peanut tissues (Figure 7). The results showed that the 26 *AhCIPK* genes had distinct tissue-specific expression patterns across the 22 tissues (leaves, stem, roots, flower, pod, and seed). *AhCIPK9* and *AhCIPK25* showed a higher expression level in leaf. *AhCIPK7* and *AhCIPK20* were mostly expressed in reproductive shoot and pattee 1 stalk; interestingly, nearly half of *AhCIPK* genes were highly expressed in reproductive organs, among which *AhCIPK2*, *AhCIPK3*, *AhCIPK10*, *AhCIPK14*, *AhCIPK15*, *AhCIPK18*, and *AhCIPK22* had strong expression in perianth; *AhCIPK3*, *AhCIPK6*, *AhCIPK8*, *AhCIPK16, AhCIPK17*, and *AhCIPK21* were highly expressed in stamens; and *AhCIPK12* and *AhCIPK26* were obviously expressed in roots. *AhCIPK11*, *AhCIPK23*, and *AhCIPK24* were highly expressed in nodules. *AhCIPK4*, *AhCIPK12*, *AhCIPK19*, and *AhCIPK24* had strong expression during the relatively later pericarp developmental stage. In addition, *AhCIPK1* and *AhCIPK19* were enriched in the earlier seed developmental stage, while *AhCIPK4* and *AhCIPK13* were expressed highly in the later seed developmental stage (Figure 7).

### Expression pattern of the *AhCIPKs* under submergence and *Ralstonia solanacearum* infection

**FIGURE 8:**
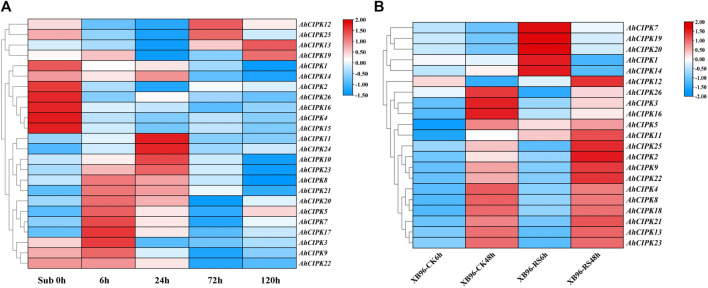
Relative expression of *AhCIPKs* under submergence and *Ralstonia solanacearum* infection. **(A)** expression characteristics of *AhCIPKs* in response to submergence at five time points (00, 06, 24, 72, and 120 h). **(B)** expression of *AhCIPKs* in response to *R. solanacearum* infection at two time points (6 and 48 h). The heatmap of the AhCIPK gene expression levels was hierarchically clustered using TBtools with the data normalization method of Z-score standardization. Legend at the right of the heatmap: red means upregulation and blue shows downregulated expression.

Plants suffer from a wide variety of environmental stressors under natural conditions. To determine the abiotic and biotic stress responses, we detected the expression of *AhCIPK* genes responding to submergence and *R. solanacearum* infection. The results showed that except the two unexpressed members *AhCIPK6* and *20*, almost all other *AhCIPK*s respond to submergence stress (Figure 8A, Supplementary Table S4)**.** *AhCIPK3*, *5*, *7*, 8, *9*, *17*, *19*, *20*, *21*, and *22* were upregulated rapidly after 6 h of the submergence treatment, while *AhCIPK10*, *11*, *23*, and *23* reached their highest expression at 24 h (Figure 8). By contrast, the expression levels of *AhCIPK1*, *2*, *4*, *14*, *15*, *16*, and *26* were inhibited under the whole submergence treatment process. We found it interesting that *AhCIPK12*, *13*, and *25* were first repressed under the earlier submergence treatment stages and were induced at the later stages. In addition, seven *AhCIPKs* (*AhCIPK1*, *5*, *7*, *11*, *14*, *19*, and *20*) were activated after 6 h *R. solanacearum* infection (Figure 8B), while six *AhCIPK* members (*AhCIPK2*, *9*, *11*, *12*, *22*, and *25*) were obviously upregulated and three (*AhCIPK3*, *16*, and *26*) were depressed after 48 h of *R. solanacearum* infection (Figure 8B, Supplementary Table S4); In total, *AhCIPK* genes might have functioned differentially in both abiotic and biotic environmental stress regulation.

### Candidate gene association of peanut *CIPK*s polymorphisms with 104 traits

**FIGURE 9:**
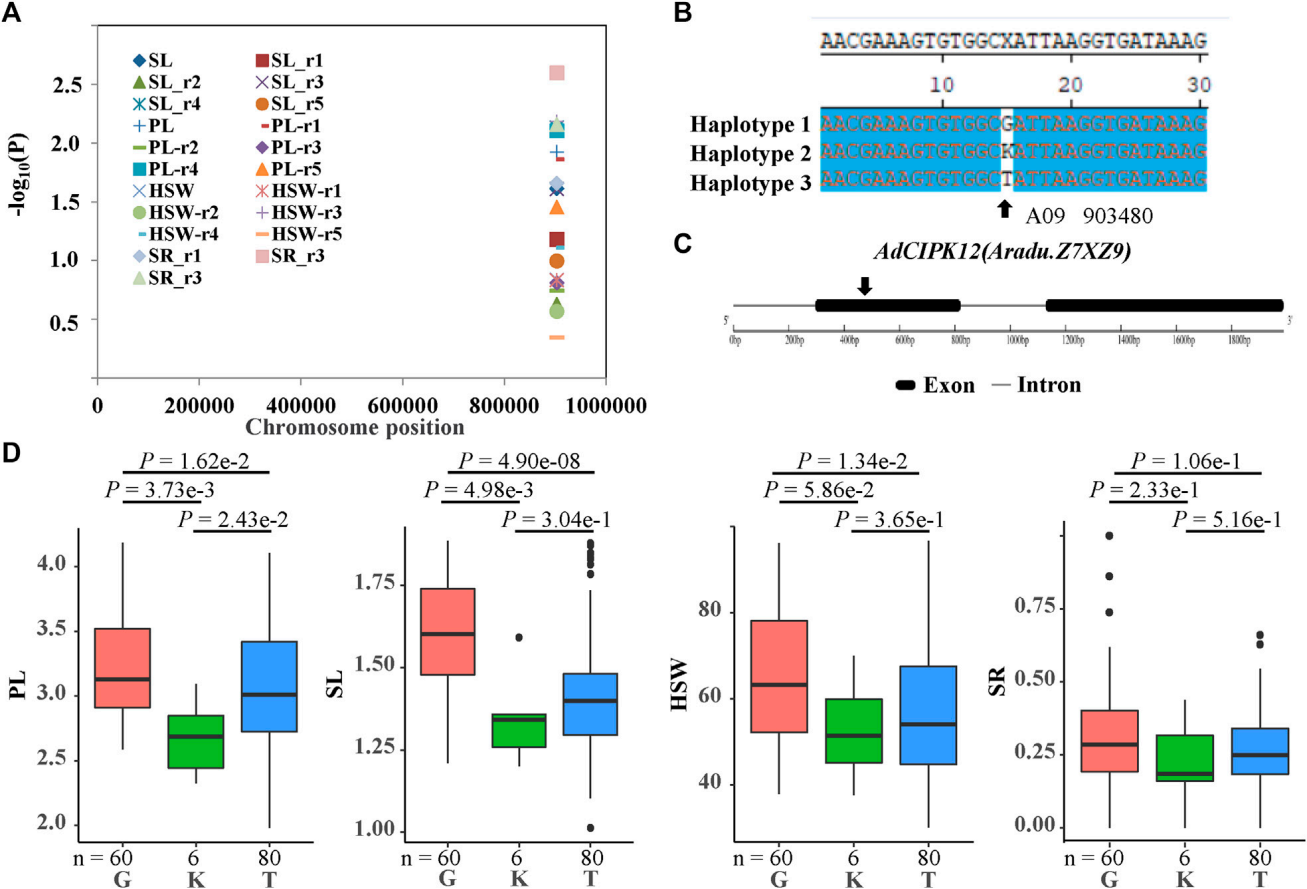
Association results and the phenotypes of the polymorphic sites of the peanut *CIPKs* associated with hundred seed weight (HSW)/pod length (PL)/ seed length (SL)/shoot root ratio (SR) variation. **(A)** association results between HSW/PL/SL/SR and the polymorphisms in peanut *CIPKs.* **(B)** sequences of the site significantly associated with HSW/PL/SL/SR variation. **(C)** gene structures of *AdCIPK12*(*Aradu.Z7XZ9*). **(D)** phenotypic comparison of haplotypes of the associated site with HSW/PL/SL/SR in five environments of the population.

To further uncover the roles of *CIPK* genes in peanut development and stress response, we performed candidate gene association analysis using 22 single-nucleotide polymorphisms in *CIPKs* from transcriptome data of 146 peanut lines and 104 phenotypes related to peanut development and stress response collected in five environments. The results indicated that one polymorphic site [A09_903480^(G/K/T)^] was significantly associated with pod length (PL), seed length (SL), hundred seed weight (HSW), and shoot root ratio (SR) traits (Figure 9A, Supplementary Tables S5–S7). Site B09_903480 mainly formed three haplotypes [B09_903480^(G/K/T)^] (Figure 9B) in the population and was located in the predicted exon region of *AiCIPK10* (Figure 9C). Results showed that PL, SL, HSW, and SR in haplotype G were significantly higher than those in haplotype T (Figure 9D).

## Discussion

### Peanut *CIPKs* did not expand with genome duplication

*CIPK* genes are widely distributed widely in the biological world; however, their number varies greatly among different species. One, two, and seven *CIPK* genes were found in green algae, *Chlorella*, and *Physcomitrella patens*, respectively (Cheong et al., 2007; Weinl and Kudla, 2009). According to previous studies, 25, 30, 27, 43, and 79 CIPK genes were identified in *A. thaliana* (125–155 Mb), rice (389 Mb), poplar (416 Mb), corn (2,400 Mb), and wheat (14,500 Mb) (Kolukisaoglu et al., 2004; Xiang et al., 2007; Chen et al., 2011; Sun et al., 2015; Zhu et al., 2021). Among *Solanaceae* plants, there were 21 and 22 *CIPK* members in tomato (Liu et al., 2017; Wang and Liu, 2018). Woody plants had 27 members (Zhang et al., 2008). This study uncovered 54 *CIPK* genes in three peanut genomes, among which 26 were from cultivated peanut (2,540 Mb). Our results support the hypothesis that the number of *CIPK* members in monocotyledonous plants is more than that in dicotyledonous plants. However, the number of peanut *CIPK* members did not expand with peanut genome size expansion.

The phylogenetic analysis revealed that *CIPK* members in *Arabidopsis*, rice, soybean, grape, and peanut could be coincidentally clustered into two distinct groups with different numbers of introns. This cluster pattern is the same as that in the previously reported *CIPK*-phylogenetic trees of *Arabidopsi*s and rice (Kolukisaoglu et al., 2004). Our results further supported the hypothesis that the ancestor of *CIPKs* evolutionarily formed in the plant genome prior to the separation of the lineages of monocotyledons and dicotyledons (Kolukisaoglu et al., 2004; Yu et al., 2007).

### Peanut *CIPK* genes functioned in stress response

The prediction of *cis*-acting elements can provide important clues for the study of gene expression regulation (Zhu et al., 2021). *Cis*-acting elements of biotic and abiotic stresses presented ubiquitously in the promoter region of peanut *CIPKs*, indicating these impact factors may interact to act on the *CIPK* regulatory mechanism (Figure 6). Compared to those in the *AhCIPKs*, the defense, stress, low-temperature response element TC-rich repeats and LTR were distributed concentratedly in several *AdCIPKs* or *AiCIPKs* in the two diploid progenitors; for example, four and three LTRs were identified in the promoter region of *AdCIPK4* and *AiCIPK11*, and four TC-rich repeats were found in the promoter region of *AdCIPK4*, indicating that the regulatory mechanism might be different between the cultivated peanut and the two diploid progenitors. Further the stress-induced expression data also elucidated the potential functions of peanut *CIPKs* in stress.

Among the orthologs between peanut and *Arabidopsis* (Table 2), the functions of the corresponding ortholog genes in *Arabidopsis* have been determined, and they functioned in influencing plant stress response (Table 2). *AdCIPK3*, *AiCIPK4*, *AhCIPK3*, and *AhCIPK16* were identified as the orthologous genes of the famous salt tolerance gene *AtCIPK24/AtSOS2* in *A. thaliana* (Halfter et al., 2000; Ishitani et al., 2000; Liu et al., 2000; Guo et al., 2001), and *AhCIPK8*, *AhCIPK21*, *AdCIPK9*, and *AiCIPK10* were found as the orthologs of *AtCIPK3* relating to the cold signal transduction (Kim et al., 2003; Pandey et al., 2008; Sanyal et al., 2017). Many other orthologs were also uncovered between peanut and the *Arabidopsis* abscisic acid-dependent and *Arabidopsis* abscisic acid-independent stress response gene *AtCIPK1* (D'Angelo et al., 2006), low-potassium tolerance gene *AtCIPK9* (Pandey et al., 2007; Singh et al., 2018; Lara et al., 2020; Kanwar et al., 2022), and cadmium stress response gene *AtCIPK11* (Liu and Guo, 2011; Zhou et al., 2015; Gu et al., 2021)*.* Therefore, these peanut *CIPK* orthologous genes may also play multiple roles in peanut stress response, especially in salt response.

### Peanut *CIPK* genes play important roles in growth and development

Many important genes were selectively expressed in specific tissues during various physiological and developmental processes (Wan et al., 2014). Our results showed that the 26 *AhCIPK* genes had distinct tissue-specific expression patterns, and several *AhCIPKs* showed higher expression level in the leaf, reproductive shoot, root, nodule, pod, and seed. We found it interesting that nearly half of *AhCIPK* genes are highly expressed in reproductive organs (Figure 7), indicating *AhCIPKs* play important roles in multiple tissue growth and development, especially the reproductive organs.

The single-nucleotide polymorphic sites in *AdCIPK12* (corresponding to *AhCIPK11*) were significantly associated with PL, SL, HSW, and SR variation. The polymorphic site in *AdCIPK12* [A09_ 903,480 ^(G/K/T)^], located in the predicted first exon region of the gene, A09_903480^(G/K/T)^, led no transition (synonymous mutation) in the peanut population. Recent studies proved that synonymous mutations also have dramatic effects on protein output (Gillen et al., 2021). These results indicated that the B09_903480 ^(G/K/T)^ sequence polymorphisms might be the actual functional sites. Further, *AhCIPK11* was mainly expressed in the nodule, peg, pericarp, and seed, especially in the middle pericarp and seed development stages, which provided additional evidence for its function in peanut pod development (Figure 6). Further investigation was needed to confirm the roles of *AdCIPK12* (*AhCIPK11*) in the pod and seed development of peanut.

## Conclusions

We definitely identified 54 *CIPK* members in cultivated and wild peanut for the first time and determined their chromosomal locations, gene structures, evolution, and expression patterns under biotic and abiotic conditions. We also focused on one gene, *AiCIPK10*/*AhCIPK21*, which was involved in pod and seed development. Our results provide valuable information for understanding the functions of the peanut *CIPK* gene family in regulating yield, quality, and stress responses in peanut.

## Data Availability

The datasets generated and analyzed during the current study are available from the corresponding author on reasonable request.
